# The therapeutic effect of Fufang Zhenshu Tiaozhi (FTZ) on osteoclastogenesis and ovariectomized-induced bone loss: evidence from network pharmacology, molecular docking and experimental validation

**DOI:** 10.18632/aging.204172

**Published:** 2022-07-12

**Authors:** Xiaojun Chen, Jiangyan Wang, Lin Tang, Qiuying Ye, Qunwei Dong, Zhangwei Li, Li Hu, Chenghong Ma, Jiake Xu, Ping Sun

**Affiliations:** 1 School of Molecular Sciences, University of Western Australia, Perth 6009, Western Australia, Australia; 2 Department of Endocrinology, The First Affiliated Hospital of Guangdong Pharmaceutical University, Guangzhou 510000, Guangdong, China; 3 College of Food and Medicine, Qingyuan Polytechnic, Qingyuan 511510, Guangdong, China; 4 Department of Orthopedic, The First Affiliated Hospital of Guangdong Pharmaceutical University, Guangzhou 510000, Guangdong, China; 5 Department of Orthopedic, Yunfu Hospital of Traditional Chinese Medicine, Yunfu 527300, Guangdong, China; 6 Department of Stomatology, The First Affiliated Hospital of Guangdong Pharmaceutical University, Guangzhou 510000, Guangdong, China; 7 School of Biomedical Sciences, University of Western Australia, Perth 6009, Western Australia, Australia

**Keywords:** Fufang Zhenshu Tiaozhi, osteoporosis, osteoclast, network pharmacology, molecular docking

## Abstract

Fufang Zhenshu Tiaozhi (FTZ) has been widely used in clinical practice and proven to be effective against aging-induced osteoporosis in mice. This study aimed to explore the mechanism of FTZ against osteoclastogenesis and ovariectomized-induced (OVX) bone loss through the network pharmacology approach. The ingredients of FTZ were collected from the previous UPLC results, and their putative targets were obtained through multiple databases. Differentially expressed genes (DEGs) during osteoclastogenesis were identified through multi-microarrays analysis. The common genes between FTZ targets and DEGs were used to perform enrichment analyses through the clusterProfier package. The affinity between all FTZ compounds and enriched genes was validated by molecular docking. The effects of FTZ on osteoclastogenesis and bone resorption were evaluated by TRAP staining, bone resorption assay and RT-qPCR *in vitro*, while its effects on bone loss by ELISA and Micro-CT *in vivo*. Enrichment analyses indicated that the inhibitory effects of FTZ may primarily involve the regulation of inflammation, osteoclastogenesis, as well as TNF-α signaling pathway. 130 pairs docking results confirmed FTZ ingredients have good binding activities with TNF-α pathway enriched genes. FTZ treatment significantly reduced TRAP, TNF-α, IL-6 serum levels and increased bone volume in OVX mice. Consistently, *in vitro* experiments revealed that FTZ-containing serum significantly inhibited osteoclast differentiation, bone resorption, and osteoclast related mRNA expression. This study revealed the candidate targets of FTZ and its potential mechanism in inhibiting osteoclastogenesis and bone loss induced by OVX, which will pave the way for the application of FTZ in the postmenopausal osteoporosis treatment.

## INTRODUCTION

Osteoporosis is a systemic bone disease characterized by deteriorated bone strength leading to increased fracture incidence [1]. Osteoporosis mainly affects the elderly population and postmenopausal women that 4–6% of people over 50 years in Europe and the United States suffer from osteoporosis, while in Asia, the affected population is more than 15% [2, 3]. Remarkably, it has been reported that the prevalence of osteoporosis-related fractures is increasing rapidly with advanced age, from 4% (women aged 50–59) to 52% (women aged over 80) [4]. In the context of the aging population, osteoporosis will become one of the major public health concerns and thus lead to a considerable economic cost in the foreseeable future.

Currently, ample evidence indicates that osteoporosis in postmenopausal women develops from bone resorption mediated by osteoclasts outweighing bone formation regulated by osteoblasts. Bisphosphonates are the mainstream first-line drugs to inhibit bone resorption [5]. Hormone therapy, such as estrogen and raloxifene, has been used in osteoporosis treatment for post-menopausal women [6]. Although useful, these anti-osteoporosis therapies have drawbacks. For instance, bisphosphonates are associated with an increased risk of atypical femur fracture, or even jaw necrosis; whereas estrogen may contribute to the increased risk of cancers, thromboembolic, and strokes [7]. Therefore, there is an urgent need for alternative and safer approaches to treating osteoporosis. Traditional Chinese medicine (TCM), a time-honored oriental medicine, possesses high clinical efficacy and less side effects and thus has been extensively used in China for millennia [8]. In East Asia, Europe, and North America, TCM has been used as an important alternative treatment [9]. Notably, accumulating *in vivo* and *in vitro* experiments have confirmed the good curative effect of TCM in treating osteoporosis [10–12].

Fufang Zhenshu Tiaozhi (FTZ) has been granted patents in China, European, and the U.S. (ZL200410051250.4, EP 2340839 B1, and US 8394431 B2). FTZ, proposed by Professor Guo Jiao, has been formulated for glucose and lipid metabolism disorders [13, 14]. It consists of 8 herbs: *Coptis chinensis* Franch., *Ligustrum lucidum* W.T.Aiton, *Cirsium japonicum* DC., *Salvia miltiorrhiza* Bunge, *Panax notoginseng* (Burkill) F.H.Chen, *Eucommia ulmoides* Oliv., *Citrus medica* L., and *Atractylodesmacrocephala* Koidz. Previous studies confirmed that FTZ possesses anti-inflammatory and anti-oxidative effects and effectively regulates glucose and lipid metabolism [13, 15]. Recently, the protective effects of FTZ on aging-induced osteoporosis has been confirmed through a metabolomic approach based on ultra-performance liquid chromatography (UPLC) to quadrupole time-of-flight mass spectrometry (UPLC-QTOF/MS). FTZ increases bone mass and strength as well as improves bone architecture of aging model mice, which may relate to its regulatory effect on sphingolipid, glycerophospholipid, and AA metabolisms [16]. All these promising findings encourage us to study further the anti-osteoporosis potential of FTZ and the possible mechanism.

Similar to other TCM prescriptions, FTZ exerts its anti-osteoporosis effect through regulating numerous targets in the human body. Therefore, it is time- and money- consuming to investigate the mechanisms of FTZ. Fortunately, with the advancement of systems pharmacology, researchers can practically investigate the pharmacological mechanisms of TCM in the system level [17]. Systems pharmacology uses integrated methods, including bioinformatics, network analysis, and information mining, to discern the potential interaction between ingredients and targets at the systemic level [18, 19].

In the current study, we used bioinformatics approaches to dissect the inhibitory effect of FTZ on osteoclast differentiation, which is an important therapeutic target of osteoporosis. Firstly, we collected the already known ingredients of FTZ through its previous UPLC study and obtained their potential targets through multiple databases. Secondly, the differentially expressed genes (DEG) related to osteoclast differentiation were obtained from GEO data sets and further matched with above FTZ targets. Thirdly, the protein-protein interaction (PPI) network was constructed, and Gene Ontology biological processes (GOBP) and KEGG pathway enrichments were performed based on the above common genes. Fourthly, the affinity between representative key compounds and osteoclast targets was validated using molecular docking. Finally, the inhibitory effect of FTZ-containing serum on osteoclast was investigated further using TRAP staining, resorption pit assay, and real-time quantitative PCR *in vitro*, while its effect on bone loss was evaluated by enzyme-linked immunosorbent assay (ELISA) and Micro-CT *in vivo*.

## MATERIALS AND METHODS

### Construction of compounds and targets database of FTZ

In order to obtain compounds that can be absorbed into blood, the ingredients of eight herbal medicines in FTZ were collected directly from our previous UPLC result using FTZ-treated rat serum [11]. Related targets are the foundations for FTZ compounds to exerting their biological functions. Thus, the TCM system pharmacology database and analysis platform (TCMSP), Swiss Target Prediction platform (STP, http://www.swisstargetprediction.ch/index.php), Similarity ensemble approach platform (SEA, http://sea.bkslab.org/) [12], and STITCH platform [20] were used to identify the active compounds of FTZ. For the STP and SEA platforms, we utilized PubChem (https://pubchem.ncbi.nlm.nih.gov/) to obtain the canonical SMILES format of related compounds.

### Identification of osteoclast differentiation-related genes

Microarray datasets, including GSE21639, GSE54779, and GSE74847 were obtained from the Gene Expression Omnibus (GEO) database using the ‘GEOquery’ package [21] in R software (4.0.2 version) [22]. These microarrays were performed to screen DEGs during osteoclastogenesis of RAW264.7 cells or monocytes under 48 h RANKL stimulation [23–25]. We used ‘Limma’ package individually [26] to obtain the DEGs between cells treated with or without RANKL for 48h in these microarrays that these DEGs may play key roles in osteoclastogenesis. *P* < 0.05 and |logfc| > 1 were set as screening thresholds to identify differentially expressed mRNAs.

Moreover, we further used the R package ‘RobustRankAggreg’ [27] to select more robust DEGs among these three arrays. Genes with |logfc| > 1 and *p* < 0.05 in the RRA analysis were considered as osteoclastogenesis-related genes. Subsequently, the heatmap was used to show the expression patterns of previous top 50 DEGs sorted by their logfc values.

### Construction of the PPI interaction network

After the above processing, the active compounds of FTZ were matched with the DEGs of osteoclastogenesis to identify the common genes that were considered as critical targets for the inhibitory effects of FTZ on osteoclast differentiation and function. These common targets were obtained by constructing the VENN diagram with the help of the ‘VennDiagram’ package. Afterward, the interaction relationship of these intersecting genes was obtained from the String database [28] that the interaction threshold was set at high confidence (0.700) to obtain credible interaction. The protein-protein interaction (PPI) network was then constructed by using Cytoscape software (3.7.2, http://www.cytoscape.org/) [29]. Furthermore, the clusters that consisted of protein families were further identified by the MCODE plugin [30]. Degree cutoff = 2, Node Score Cutoff = 0.2, and K-Core = 2 were set as criteria to determine these important gene modules.

### Biological process and pathway enrichment analysis

Above DEGs related to osteoclastogenesis were further used to conduct the Gene Ontology biological process enrichment (GOBP) and Kyoto Encyclopedia of Genes and Genomes (KEGG) pathways analysis through the ‘clusterProfiler’ package [31]. The cut-off *P* value of these enrichment terms was set to less than 0.05. These enriched biological processes and pathways may contribute to the inhibitory effect of FTZ on osteoclastogenesis.

### FTZ ingredients-targets docking

To a certain extent, molecular docking method can predict binding mechanisms and validate the interactions between active ingredients and target proteins. The RCSB Protein Data Bank (https://www.rcsb.org/) was used to obtain the 3D formats of interested protein targets. Consequently, the ingredients of FTZ were download from the PubChem database(https://pubchem.ncbi.nlm.nih.gov/) and converted into the mol2 format using ‘OpenBabel’ software (version 3.1.1) [32]. And then DeepSite [33] was utilized to determine protein docking regions. Next, the binding affinity between ingredients and their targets was calculated by Autodock vina (version 1.1.2). Affinity ≤ −4.25 kcal/mol, ≤ −5.0 kcal/mol, and ≤ −7.0 kcal/mol indicates possible binding, good binding, and strong binding strength between ligands and receptors, respectively [34]. Finally, PyMOL™ (version 2.5.2) and Discovery Studio 2020 were used to generate the 3D and 2D graphs for the docking results.

### Cell culture and treatment

FTZ was provided by the first affiliated hospital of Guangdong Pharmaceutical University. All *in vivo* experiments were approved by The Animal Ethics Committee of Guangdong Pharmaceutical University (Number: GYFYDW2021007). FTZ was diluted with 0.9% normal saline (NS) in a 1 g/mL concentration. According to FTZ clinical oral dose, rats (*n* = 2) were gastrically administered 1.55 g/kg/d FTZ, once a day for 7 consecutive days. The setting of concentration of FTZ was determined by the conversion from FTZ’s dose in clinical use according to the commonly applied practice guide [35]. Rats in the control group were given the same dose of NS. Rats were anesthetized with 3% isoflurane before cervical dislocation. After sacrificed, blood was obtained from the abdominal aorta 1 h after FTZ administration on day 7, and then centrifuged at 3000 r/min for 10 min. Subsequently, serum was heated at 56°C for 30 min, filtered with a 0.22 *μ*m micropore filter, and stored at –20°C for further experiments.

The tibia and femur of C57BL/6J mice aged 4–6 weeks were used to isolate bone marrow macrophages (BMMs). Mice were anesthetized with 2% isoflurane and then sacrificed using cervical dislocation. After resuspended with the prepared Alpha-Minimum essential medium (containing 10% fetal bovine serum (FBS), 1% penicillin–streptomycin and 1% glutamine), BMMs were planted into a 35 mm culture dish and cultured overnight at 37°C an incubator with 5% CO_2_. The next day non-adherent cells were collected and planted into 24 well culture plates with a density of 1 × 10^6^ cells/well. Using medium containing M-CSF (25 ng/mL, R&D Systems, USA) and 10% rat serum, BMMs were assigned to control, and FTZ groups. After 72 h, BMMs were further incubated with the medium containing 25 ng/mL M-CSF, 30 ng/mL RANKL (R&D Systems, USA), 10% FTZ-containing serum, and control serum for 8 days.

### Cell proliferation

For the cytotoxicity assay, BMMs were planted in 96-well plates using a density of 6 × 10^3^ cells/well. After cells adhered, they were treated with FTZ or control serum for 48 h. After that, MTS solution (20 μL per well) was added, and cells were then incubated for an additional 2 h. The optical density (OD) was measured by spectrophotometric absorbance at 490 nm using a microplate reader (Thermo Fisher Scientific, USA).

### TRAP staining and resorption pit assay

After 8 days of intervention, BMMs were washed with deionized water, fixed with 4% (wt/vol) paraformaldehyde, and then stained with a TRAP staining kit. The images of stained cells were captured using an inverted light microscope (Leica, Germany). BMMs were seeded in Corning Osteo Assay Plate at a density of 1.5 × 10^6^ cells/well and treated with the above processes. TRAP staining kit and Corning Osteo Assay Plate 3987 were purchased from Sigma, USA. After 10 days treatment, the culture medium was discarded, and 200 μL/well 10% bleaching water was added. Cells were incubated for 5 min in the dark, washed with pure water 3 times, and dried at room temperature. All wells were observed by an inverted light microscope.

### Real-time quantitative PCR analysis

Total RNA was extracted after 8 days of intervention according to the instructions from a Takara Mini BEST Universal RNA extraction kit (Takara, Japan), and RT-qPCR was carried out in a real-time PCR system (Thermo Fisher Scientific, USA). Specific sequences of PCR were amplified for 1 min at 55°C followed by 45 cycles of 95°C (30 s) and finally dissociated for 34 s at 60°C. Data were normalized to the expression levels of GAPDH, and then calculated using the ΔCT method. Specific primers used in PCRs are shown in Table 1.

**Table 1:** Primer sequence table.

**Gene**	**Sequence (5′–3′)**
GAPDH	Forward: TGTAGACCATGTAGTTGAGGTCA
	Reverse: AGGTCGGTGTGAACGGATTTG
Trap	Forward: ACCATTGTTAGCCACATACG
	Reverse: GTGAAACCGCAAGTAGCC
Mmp9	Forward: AGTTTGGTGTCGCGGAGCAC
	Reverse: TACATGAGCGCTTCCGGCAC
Nfatc1	Forward: GGGTCAGTGTGACCGAAGAT
	Reverse: GGAAGTCAGAAGTGGGTGGA

### Mouse ovariectomy experiment

Twelve C57BL/6J female mice (11-weeks-old; 18.23 ± 0.11 g) were acquired from the Animal Center of Guangdong Pharmaceutical University. These mice were divided into sham group, ovariectomy (OVX) group, and OVX group treated with 1.55 g/kg FTZ (*n* = 4) randomly and fed adaptively for one week. Under isoflurane anesthesia, bilateral ovariectomy was carried out to induce osteoporosis in the OVX and OVX+FTZ groups, while in the sham group, mouse ovaries were gently raised without resection. After one week of recovery, mice in the OVX+FTZ group were gastrically administered 1.55 g/kg/d FTZ, once a day for 12 weeks. Mice in the Sham and OVX groups were administered with the same dose of NS as a control.

### Micro-CT analyses and ELISA

After sacrifice, the surrounding muscles, ligaments, and other soft tissues were removed, femur specimens were fixed in 10% neutral-buffered formalin. The prepared bone was transferred to a moderate resolution Inveon Micro-CT scanner (Siemens Inveon™, Munich, Germany). The scan setting parameters were as follows: an isometric resolution of 33 μm, with X-ray energy settings of 80 kV and 500 μA. A 3D reconstruction was generated. We identified the proximal femur as the region of interest (ROI). Specifically, the volume began at the bottom of the growth plate at a distance of 0.5 mm, and the total distance was 1 mm. Several parameters of trabecular bone within the ROI, including bone volume/total volume (BV/TV), bone surface/bone volume (BS/BV), trabecular number (Tb.N), and trabecular separation (Tb.Sp) were identified by a constant threshold. The Micro-CT scanning, selection and software analysis were performed by the same operator, who was blinded to the experimental contents.

The serum levels of E2, TRAP, TNF-α, and IL-6 in each group were determined by ELISA in strict accordance with the instructions.

### Statistical analysis

R software (4.0.2 version) was used to conduct all the statistical analyses. Student’s *t*-test was used to compare with two-groups data. *P* < 0.05 was designated a statistically significant difference.

## RESULTS

### FTZ targets prediction and analysis

**Figure 1:**
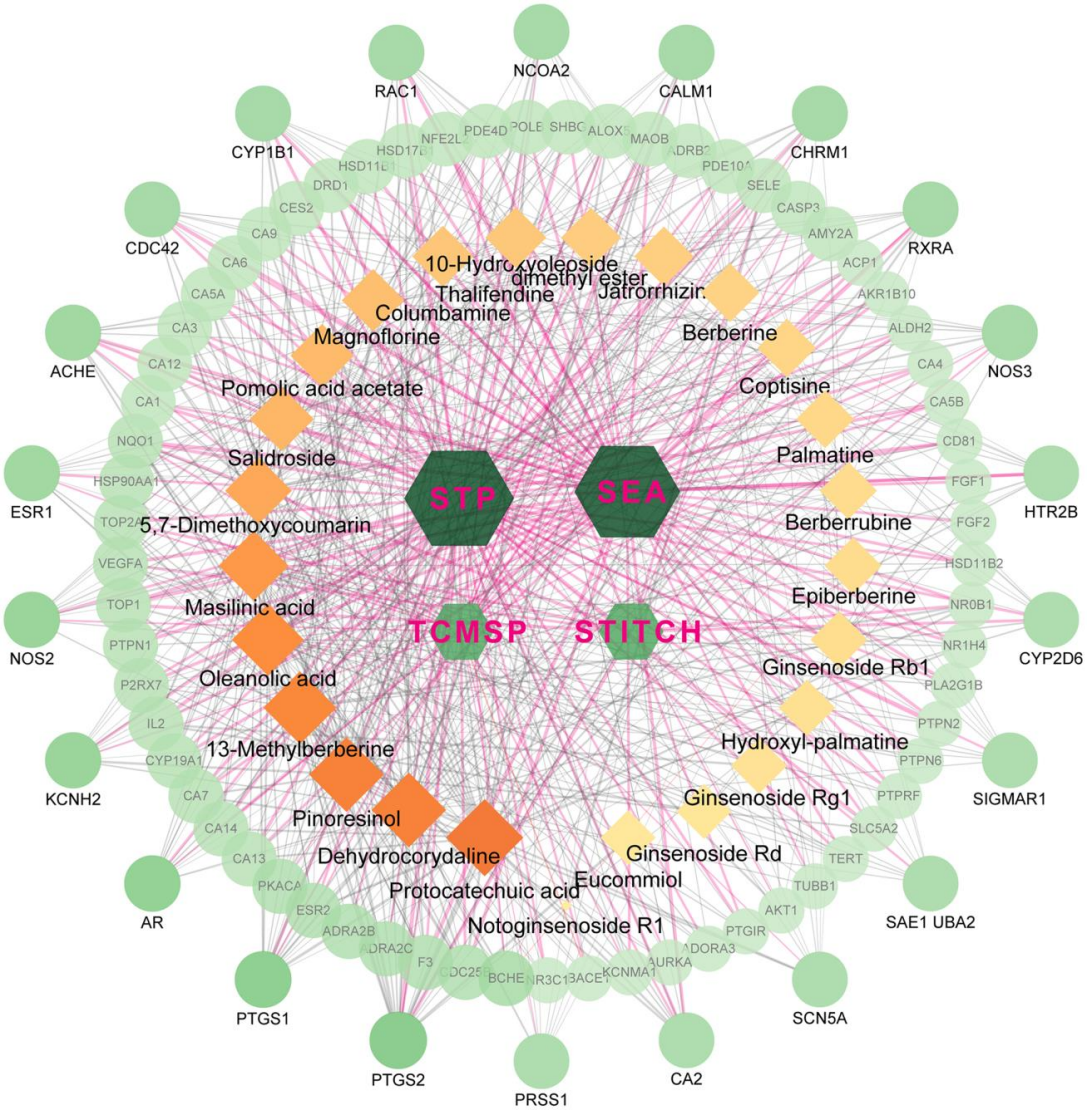
**The network for database-compound-target connection.** This network consists of 623 nodes and 1870 edges. The hexagon nodes represent the databases, rectangles represent the compounds and round ones represent targets. All nodes’ area and color changes are shown according to their degree value, and only gene nodes whose degree greater than 5 are presented for better clarity and concise presentation. Light purple lines represent the database-target interactions.

The active ingredients after oral administration of FTZ were identified by UPLC/Q–TOF–MS. Twenty-seven compounds originated from FTZ were confirmed and one of which could not be recognized. Thus, the 26 remaining compounds were collected for further analysis, including 10-Hydroxyoleoside, dimethyl ester, Salidroside, Hydroxylpalmatine, Protocatechuic acid, Magnoflorine, Eucommiol, Thalifendine, Columbamine, Epiberberine, Coptisine, Jatrorrhizine, Notoginsenoside R1, Berberrubine, Palmatine, Berberine, Ginsenoside Rg1, De-hydrocorydaline, 13-Methylberberine, 5,7-Dimethoxycoumarin, Ginsenoside F1, Ginsenoside Rb1, Ginsenoside Rd, Pinoresinol, Pomolic acid acetate, Oleanolic acid, and Maslinic acid. Through target fishing, 593 predicted targets were obtained using TCMSP, SEA, STITCH, STP databases. Among the 26 ingredients, Protocatechuic acid has the highest number of targets (142 targets), while no target was identified for Ginsenoside F1. Therefore, the databases-compounds-targets network of FTZ consists of 623 nodes (4 in databases, 26 in bioactive ingredients, 593 in potential targets) and 1870 edges. For better clarity and concise presentation, only target nodes whose degree is greater than 5 were shown (Figure 1). Detailed information can be found in the Supplementary Data.

### Differential expression analysis for osteoclastogenesis

The differential expression analysis of GSE21639 identified 499 DEGs related to osteoclastogenesis, including 203 upregulated genes and 296 downregulated ones. GSE54779 array has 745 DEGs which consist of 474 upregulated genes and 271 downregulated ones. Moreover, there are 463 upregulated and 273 downregulated genes identified in the GSE74847 DEGs.1980 osteoclast DEGs were obtained from three arrays, and 1733 genes remained after deduplication.

**Figure 2:**
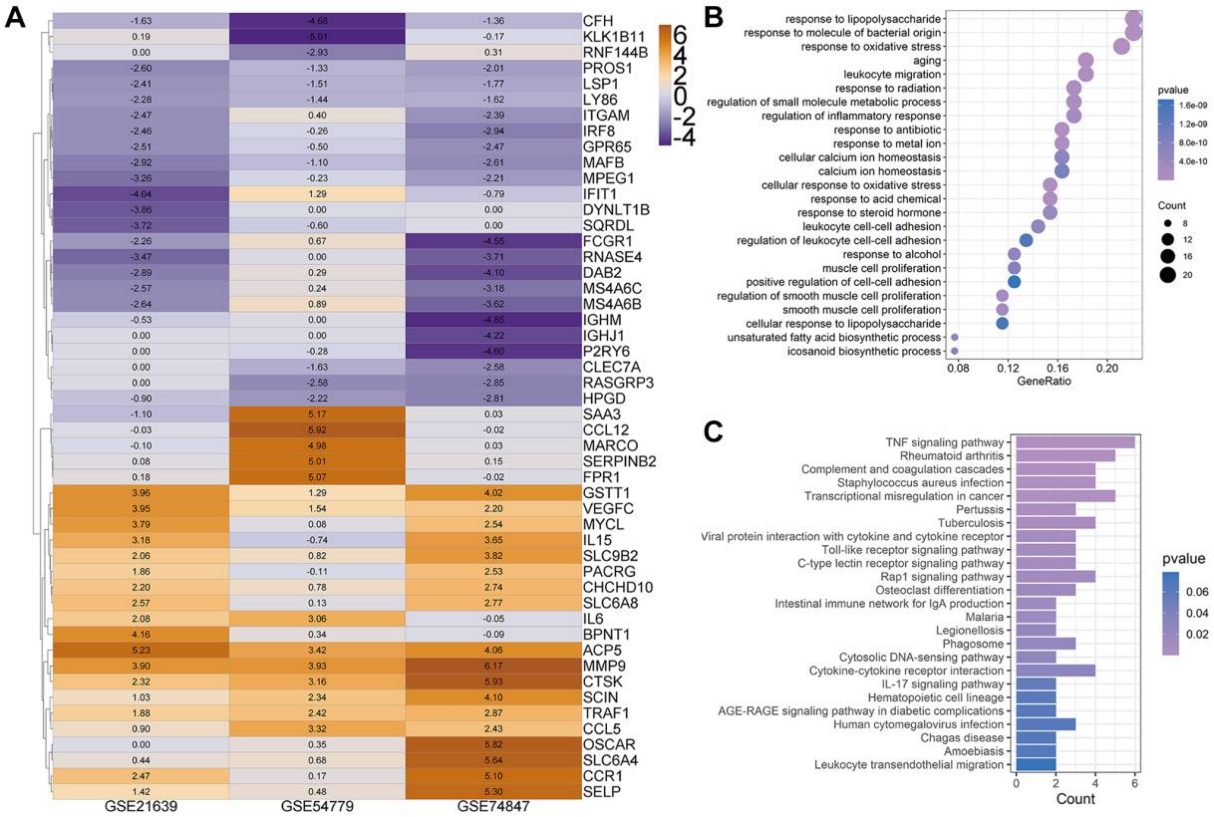
**Heatmap for top 50 differentially expressed genes (DEGs).** (**A**) Top 50 DEGs of osteoclast differentiation obtained from Robust rank aggregation (RRA) analysis. The color of the cells ranging from yellow to purple represents the logfc value of genes ranging from high to low. (**B** and **C**) The dot plot and bar plot show the enrichment results of biological process (**B**) and KEGG from total osteoclast DEGs based on RRA results (**C**).

To investigate the critical biological processes and pathways, we further used the Robust rank aggregation (RRA) method to fitter out solid genes which severed as DEGs in each array. 52 DEGs (|logfc| ≥ 1, *P* < 0.05) were identified, 25 of which were upregulated and 27 downregulated during osteoclastogenesis (Supplementary Table 1). A clustering diagram was constructed to display the top 50 RRA DEGs sorted by their absolute logfc values. The color depth changes according to their logfc values presented inside the cells (Figure 2A). Osteoclastic marker genes, such as Mmp9 and Acp5, are significantly increased among these three arrays, and these results proved that our analyses are accurate and reliable.

The GOBP enrichment of these 52 DEGs was conducted through the clusterProfiler package, and then a total of 126 BP terms were enriched. After sorting these terms in ascending order of *P* value and gene counts, the top 25 BP terms were displayed in Figure 2B. Several BP terms closely related to osteoclastogenesis or OC functions were identified, such as the regulation of inflammatory response (1st, GO:0050727 and *P* < 0.0001), myeloid cell differentiation (6th, GO:0030099 and *P* < 0.0001), osteoclast differentiation (GO:0030316, 26th and *P* < 0.0001), bone resorption (GO:0045453, 44th and *P* < 0.0001) and cellular calcium ion homeostasis (GO:0006874, 54th and *P* < 0.0001). Moreover, several KEGG terms closely related to osteoclastogenesis were also enriched (Figure 2C), such as TNF-α signaling pathway (hsa04668, 1st and *P* < 0.0001) and Osteoclast differentiation (hsa04380, 12th and *P* < 0.0001).

### Intersection targets and PPI network

**Figure 3:**
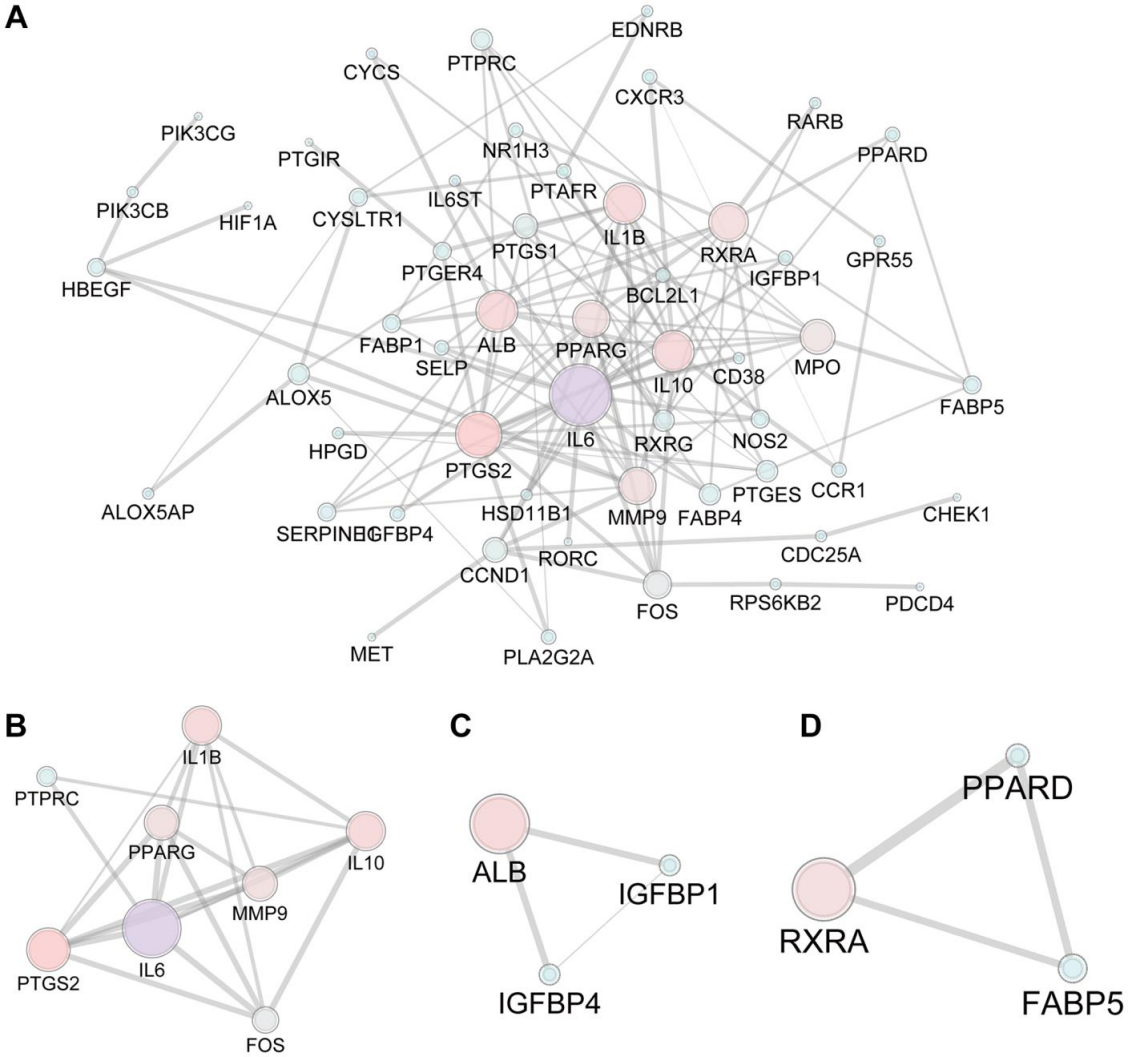
**The PPI network of 77 candidate targets of FTZ in inhibiting osteoclast differentiation.** (**A**) All nodes’ area and color changes are shown according to their degree value. (**B**–**D**) Key submodules from 77 candidate targets analyzed by the MCODE plug-in of Cytoscape software. All nodes’ area and color changes are shown according to their degree value.

The 593 predicted targets of FTZ were matched with 1980 osteoclast DEGs (1733 unique DEGs) obtained from three arrays, resulting in the intersection of 77 composite targets between FTZ and osteoclast-related genes (Supplementary Table 2). These common targets may be the therapeutic genes for the inhibitory effect of FTZ on osteoclastogenesis (Figure 3). Subsequently, we analyzed the PPI between 77 common genes based on the result from the STRING database. This PPI network, consisting of 53 nodes and 122 edges, was visualized by Cytoscape (Figure 3A). Based on the area, color depth, and location of nodes, Il6, IL10, Mmp9, Ptgs2 and Il1β may play key roles in this network. Three significant modules were identified by using MCODE (Figure 3B–3D). Significantly, the core genes of module A (Figure 3B), Il6 and Mmp9, are the critical DEGs among 52 RRA genes.

### GOBP and KEGG pathway enrichment analysis

**Figure 4:**
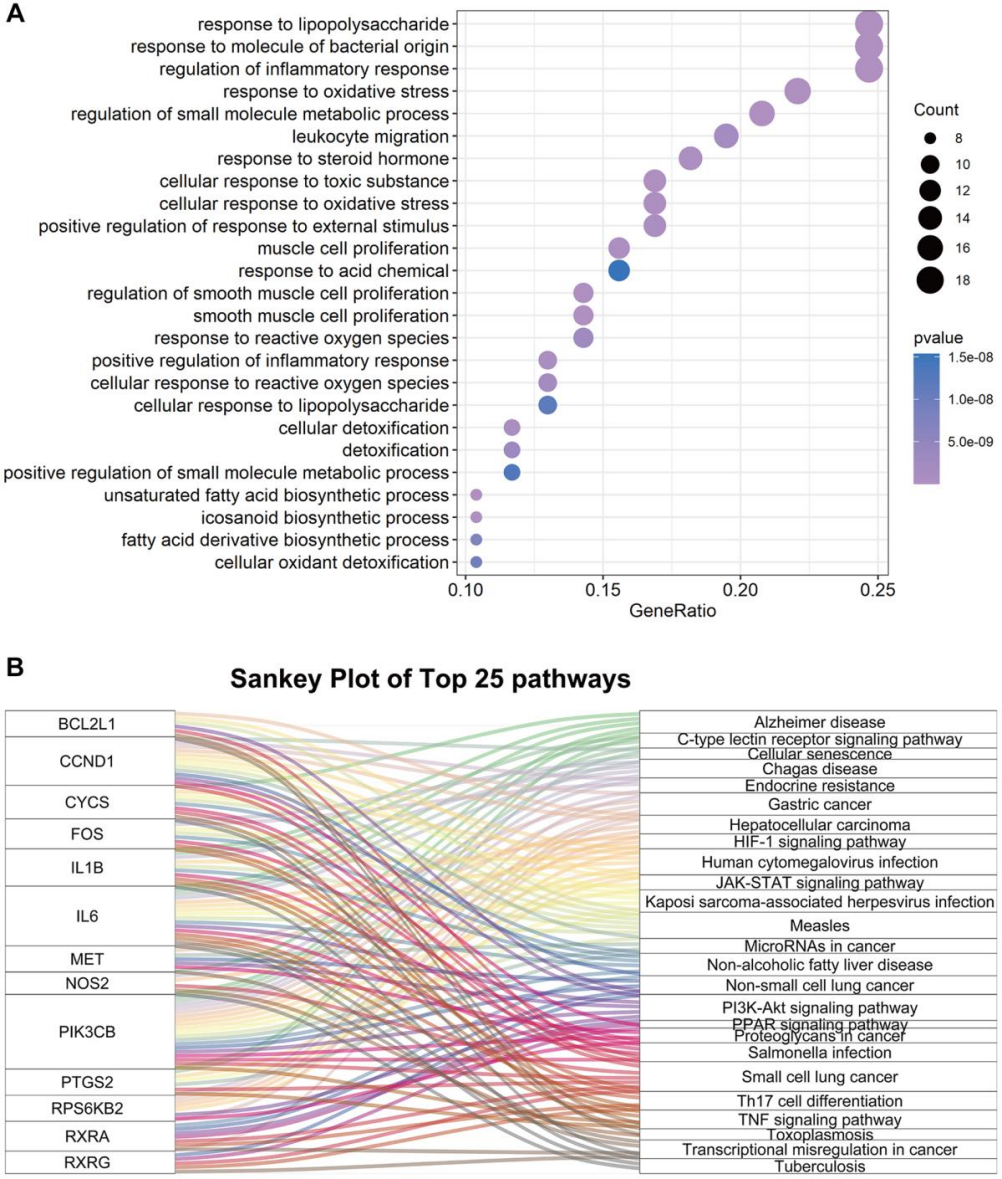
**The enrichment results of 77 common genes between FTZ pharmacological target genes and osteoclast-related genes.** (**A**) Top 25 terms of the biological process enrichment. (**B**) Sankey diagram for the top 25 KEGG enrichment terms, displayed the relationship between enriched genes and pathway terms.

To further explore the potential mechanisms of FTZ in suppressing osteoclastogenesis, GOBP enrichment of these 77 targets was conducted through the clusterProfiler package, and then a total of 1300 BP terms were enriched. After sorting these terms in ascending order of *P* value and gene counts, the top 25 BP terms were displayed in Figure 4A. Among these top 25 BP terms, the regulation of inflammatory response (GO:0050727, 3rd and *P* < 0.0001) and the response to oxidative stress (GO:0006979, 4th and *P* < 0.0001) are all proven critical processes related to osteoclastogenesis. It is noteworthy that these BP terms have been found to be associated with osteoclastogenesis in the RRA analysis part (Figure 2B). Moreover, the terms, including the regulation osteoclast differentiation (GO:0030316, 771th and *P* < 0.01), myeloid cell homeostasis (GO:0002262, 589th and *P* < 0.01) and myeloid cell differentiation (GO:0030099, 770th and *P* < 0.01), were identified in the total BP enrichment.

Next, KEGG enrichment was performed to investigate the regulating mechanisms of FTZ in osteoclastogenesis on the pathway level. The results showed that 77 targets were enriched to the 93 signaling pathways (*P* ≤ 0.05). Then, we sorted the top 25 pathways to build up a Sankey plot (Figure 4B), which demonstrated that Il1β, Il6, Pik3cb, Ptgs2, Bcl2l1, Ccnd1, Cycs, Fos, Met, Nos2, Rps6kb2, Rxra, and Rxrg are the most frequently enriched genes that may play critical roles in the therapeutic effect of FTZ. Moreover, TNF-α signaling pathway (hsa04668), related to osteoclastogenesis, is one of the top 25 pathways. A pathway related osteoclast differentiation (hsa04380, 70th and *P* < 0.05) was also identified.

### Validation of compound-target interaction

**Figure 5:**
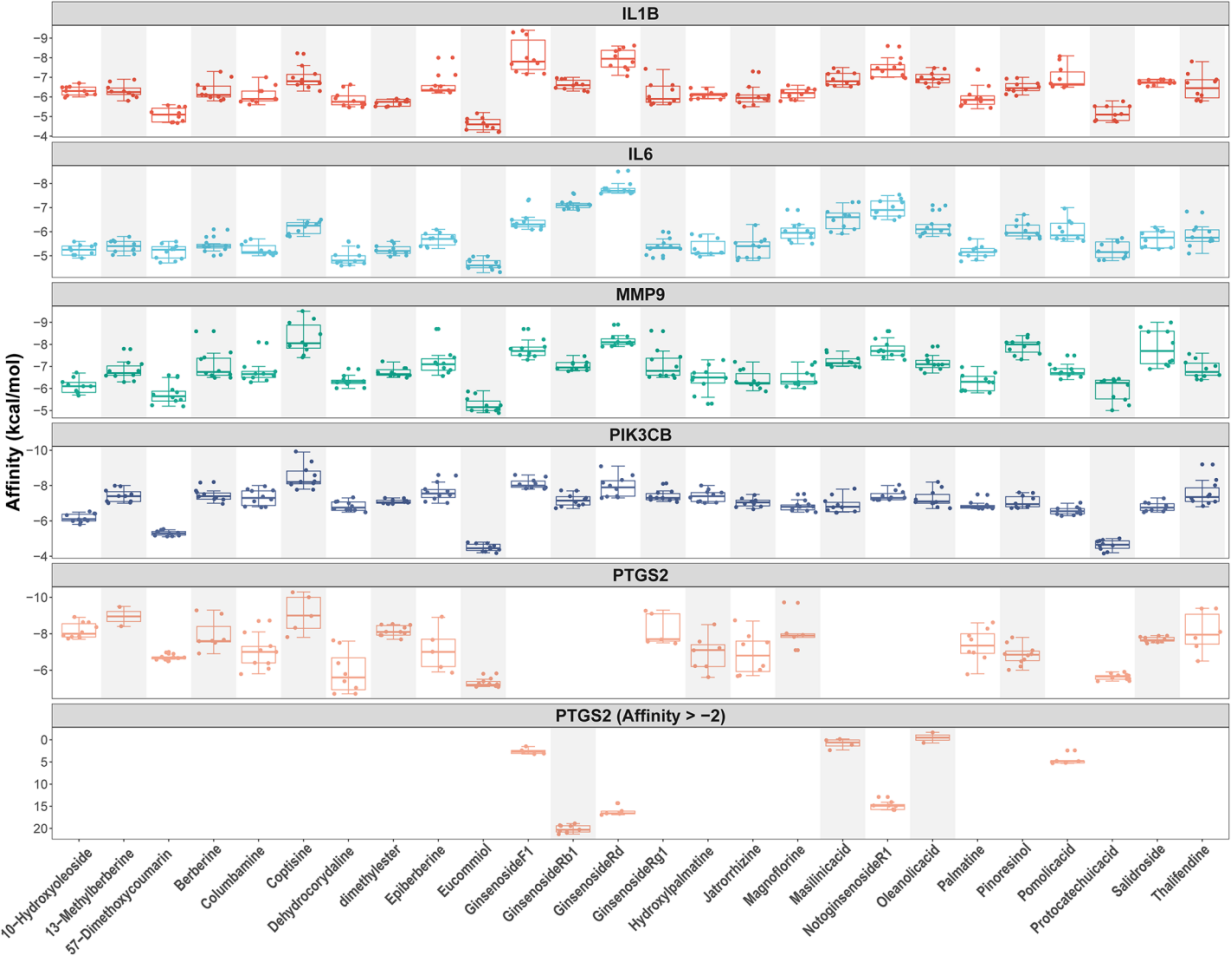
**Docking models between all compounds and enriched genes of the TNF pathway are shown as boxplot.** Each point represents the affinity of docking pose between a compound (ligand) and an enriched gene (receptor).

**Figure 6:**
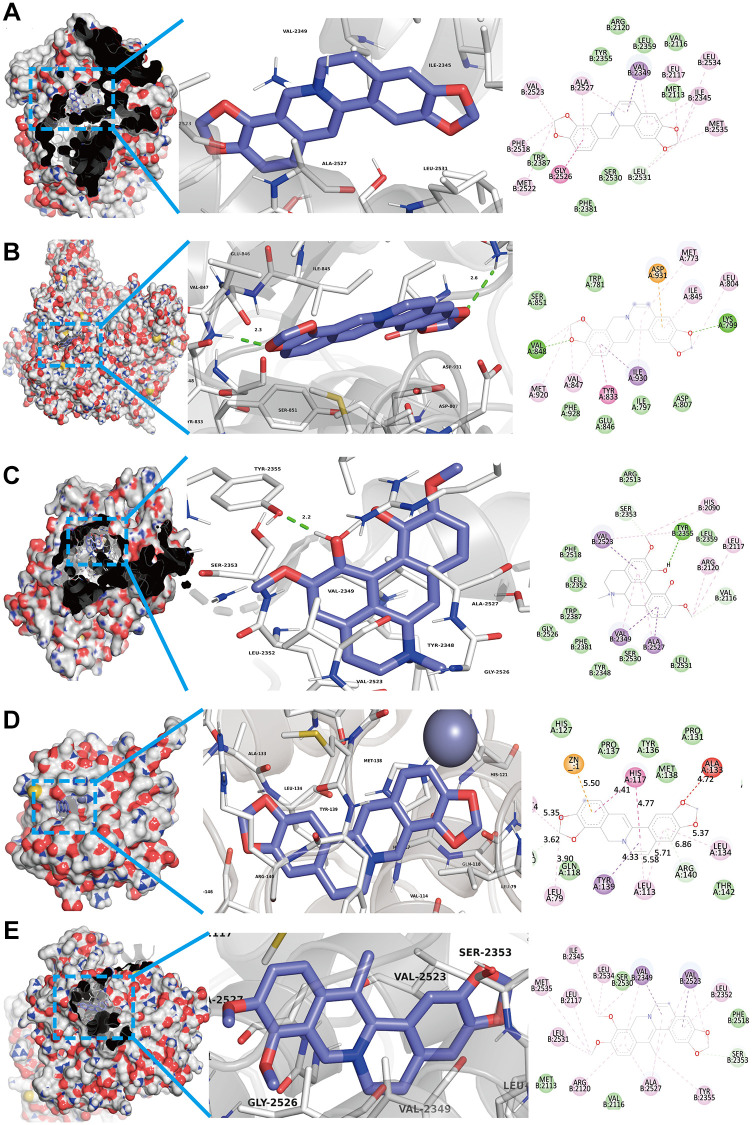
**Top 5 docking modes between compounds and enriched genes of the TNF pathway are shown as 3D and 2D diagrams.** (**A**) The binding modes of Coptisine to Ptgs2. (**B**) The binding modes of Coptisine to Pik3cb. (**C**) The interaction modes of Magnoflorine with Ptgs2. (**D**) The binding modes of Coptisine to Mmp9. (**E**) The interaction modes of 13-Methylberberine with Ptgs2.

As shown in Figures 2C and 4B, we confirmed that TNF-α pathway plays a key role in osteoclastogenesis and may be a putative therapeutic mechanism for FTZ inhibiting osteoclastogenesis. Therefore, we further investigated the combination activity between 27 FTZ ingredients and 6 enriched genes of TNF-α pathway, including Ptgs2, Pik3cb, Mmp9, Il1β, Il6, and Fos. As we cannot predict any docking pocket for Fos in DeepSite, we only analyzed another five receptors and obtained 130 pairs docking results after mass molecular docking using AutoDock Vina (Supplementary Table 3). It was observed that 63.8% of the docking complexes had binding energies ≤ −7.0 kcal/mol, 93.8% complexes ≤ −5.0 kcal/mol, with their targets (Figure 5). These results indicated FTZ components have good binding activities with TNF-α pathway enriched genes. It is noteworthy that Ginsenoside F1, Rb1, Rd have higher binding affinity with Mmp9, IL1β, IL6 proteins compared to others, which implied that these ingredients may play critical roles in the therapeutic effects of FTZ. To a certain extent, these docking results confirmed the reliability of our current network pharmacological analysis. Moreover, the PyMoL and Discovery Studio software were utilized to visualize the top 5 docking complexes (Figure 6 and Table 2).

**Table 2:** Top 5 docking results between compounds and enriched genes of TNF pathway.

**Compounds**	**Targets**	**PDB ID**	**Affinity (kcal/mol)**
Coptisine	Ptgs2	1CVU	−10.3
Coptisine	Pik3cb	2Y3A	−9.9
Magnoflorine	Ptgs2	1CVU	−9.7
Coptisine	Mmp9	6ESM	−9.5
13-Methylberberine	Ptgs2	1CVU	−9.5

### The inhibitory effect of FTZ on osteoclastogenesis *in vitro*

**Figure 7:**
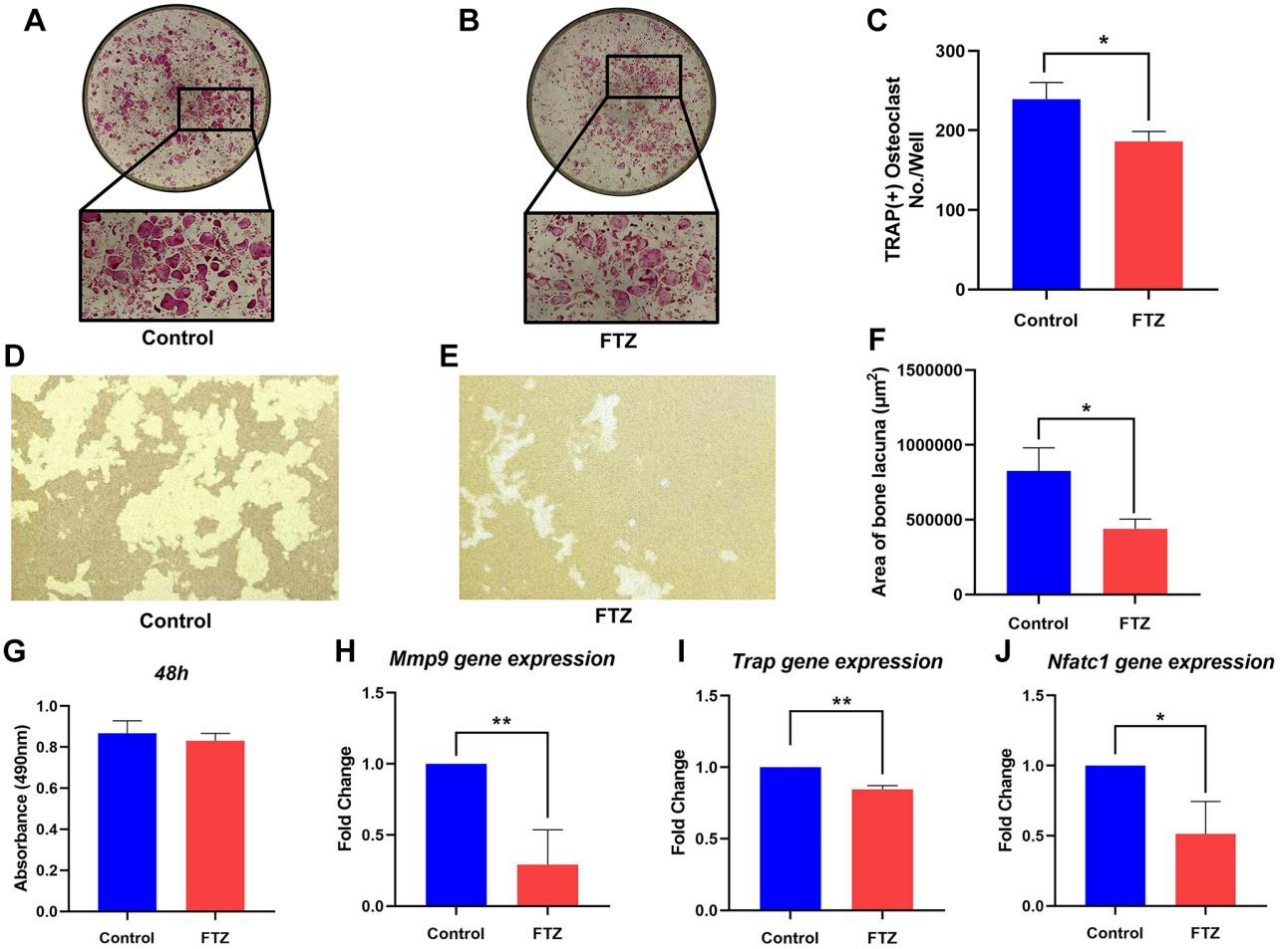
**FTZ suppresses the function of osteoclasts and osteoclast-specific genes expression.** (**A** and **B**) TRAP staining for osteoclast stimulated by RANKL (Control) and incubated with FTZ-containing serum (FTZ), respectively. (**C**) The number of the TRAP-stained osteoclasts (>3 nuclei) was calculated. (**D** and **E**) Representative images of eroded areas on hydroxyapatite-coated plates under RANKL or FTZ treatments (magnification = 10×). (**F**) Quantitative analysis of pit area with or with FTZ treatment. (**G**) BMMs were treated with FTZ for 48 h and the MTS assay was used to measure cell viability. (**H**–**J**) Determination of key mRNA expression during osteoclast differentiation by RT-qPCR, including Mmp9, Trap, Nfatc1 (*n* = 3). ^*^p < 0.05, ^**^p < 0.01.

To investigate the effect of FTZ on osteoclast differentiation, BMMs were induced by RANKL and treated with FTZ-containing serum. After 8 days RANKL induction, TRAP staining was performed and observed under a microscope. Figure 7A shows that BMM cells differentiated into mature osteoclasts under the RANKL stimulation. However, this differentiation can be significantly suppressed by FTZ serum (Figure 7B and 7C). And FTZ serum was found to have no cytotoxicity on BMMs according to the MTS assay results (Figure 7G).

Resorption pit assay was conducted to observe the inhibition of FTZ to osteoclast-induced bone resorption under RANKL stimulation. Mature osteoclasts diverged from BMMs under RANKL and M-CSF induction had strong bone resorption capacity. Their bone-resorbing activity was significantly attenuated under the intervention of FTZ serum (Figure 7D–7F). RT-qPCR was further used to confirm the inhibitory effect of FTZ on osteoclastogenesis at the gene expression level. The expression levels of Mmp9, Trap, Nuclear factor of activated T-cells, cytoplasmic 1 (Nfatc1) were found to be remarkably reduced in the FTZ group (Figure 7G–7J).

### FTZ attenuated ovariectomy-induced bone loss *in vivo*

**Figure 8:**
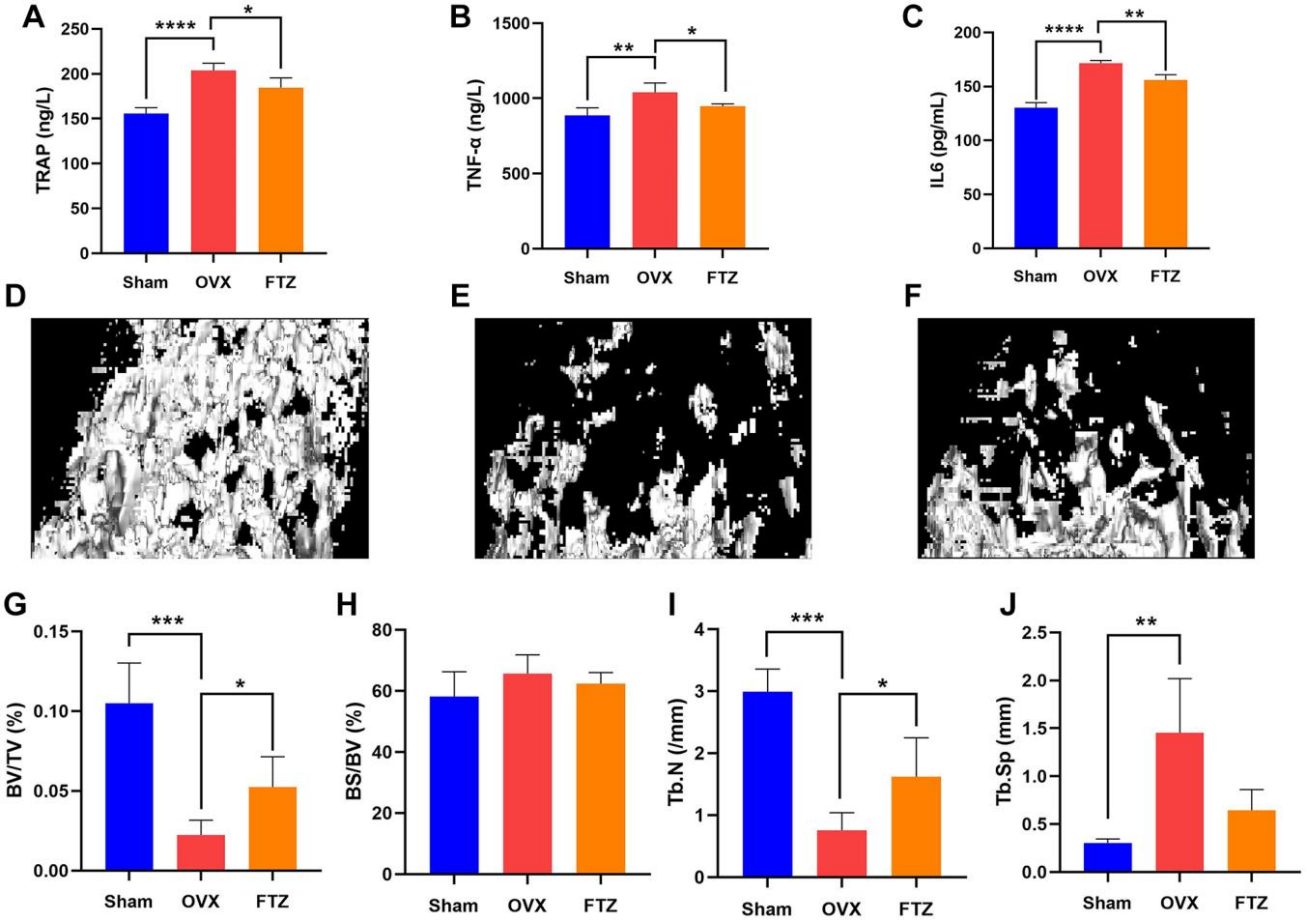
**FTZ treatment improves ovariectomized (OVX)-induced bone loss *in vivo*.** (**A**–**C**) Serum levels of TRAP, TNF-α, IL6 in different groups were detected by ELISA. (**D**–**F**) Representative μCT images showing the bone mass of Sham, OVX, FTZ groups, and indicating that the bone loss was prevented by FTZ treatment. (**G**–**J**) μCT quantitative parameters for bone microstructure including BV/TV, BS/BV, Tb.N, and Tb.Sp (*n* = 4 per group). ^*^p < 0.05, ^**^p < 0.01, ^***^p < 0.001. Abbreviations: BV/TV: bone volume per tissue volume; BS/BV: bone surface per bone volume; Tb.N: trabecular number; Tb.Sp: trabecular spacing.

To further assess the therapeutic effect of FTZ on osteoclastogenesis *in vivo*, we established OVX mice model. After 12 weeks of treatment, the mice were sacrificed, and their left femurs and serum were used for further analysis by Micro-CT and ELISA (Figure 8). Serum E2 levels of mice in the OVX group were significantly decreased compared with the sham group, while in the FTZ group they were significantly higher compared to the OVX group (Figure 8A). Moreover, FTZ treatment was able to reverse the increased serum levels of TRAP, TNF-α, and IL-6 induced by OVX (Figure 8A–8C).

The reconstructed 3D images confirmed that OVX mice underwent significant bone loss, which was attenuated by FTZ treatment (Figure 8D–8F). In the quantitative analysis, BV/TV and Tb.N were significantly increased with FTZ treatment. Although there were no differences for BS/TS and Tb.Sp between the OVX and the FTZ groups, FTZ treatment was able to partly improve the increased trabecular spacing induced by OVX (Figure 8G–8J).

## DISCUSSION

Osteoporosis is a chronic bone disease, and its features include weak bone tissue and increased risks of fracture. Considering the increased aging global population, osteoporosis has been considered as a common disease threatening elderly and postmenopausal people. Therefore, it is economical and feasible to take effective treatments to prevent osteoporosis and its fracture complications. The quantitative and qualitative deterioration of bone tissues caused by osteoporosis results from an imbalance between bone resorption dominated by osteoclast and bone formation by osteoblasts [36, 37]. In addition, inflammatory response also plays a key role in regulating bone remodeling in osteoporotic condition [38]. After menopause, estrogen deficiency increases the expression of pro-inflammatory and pro-osteoclastic cytokines such as TNF-α [39].

In recent years, developing novel drugs from TCM has attracted attention in the context of finding alternative and safer measures capable of inhibiting osteoporosis [40, 41]. FTZ has been widely used in clinical practice and has recently proven to be effective against aging-induced osteoporosis in mice [16]. Previous studies also confirmed that FTZ possesses anti-inflammatory and anti-oxidative effects and effectively regulates glucose and lipid metabolism [13]. Given the complexity of compounds, FTZ may exert its pharmacological activities with multiple protein targets. Therefore, it is difficult to explore the anti-osteoporosis mechanism of FTZ. Fortunately, network pharmacology can serve as a comprehensive and powerful tool to investigate the complicated mechanisms of TCM [42]. In the current study, we utilized network pharmacology method to explore the pharmacological mechanism of FTZ on osteoclastogenesis, following *in vitro* and *in vivo* validation.

After constructing the databases-compounds-targets network (Figure 1), we noticed that the top 5 compounds, including Protocatechuic acid, Dehydrocorydaline, Pinoresinol, 13-Methylberberine, and Oleanolic acid, may play critical roles in this network. Indeed, Protocatechuic acid has been found to inhibit osteoclastogenesis and attenuate bone loss in OVX mice through down-regulating osteoclast specific gene expression (Nf-κb, Mmp9, Ctsk, Trap and Nfatc1). Further, MAPK signaling and inflammatory proteins such as NF-κB and Cox-2 expressions were also significantly down-regulated by protocatechuic acid treatment [43, 44]. Similarly, Oleanolic acid possesses the bone protective effects in OVX mice by inhibiting osteoclastogenesis. Oleanolic acid can effectively decrease the RANKL-induced expression of osteoclast specific genes, including Mmp9, Ctsk, and Trap [45, 46]. Moreover, other compounds in this network also have been proven able to suppress osteoclast differentiation and bone-resorbing activity, including Maslinic acid [47], Salidroside [48], Ginsenoside Rb1 [49], Ginsenoside Rg1 [50], Berberine [51], Palmatine [52], Notoginsenoside R1 [53], Jatrorrhizine [54], Coptisine [55] and Magnoflorine [56]. These active compounds may be the cornerstones for the promising anti-osteoporosis effect of FTZ.

After constructing a Venn diagram between FTZ targets and DEGs of osteoclast differentiation, 77 common genes were identified as therapeutic targets which may play critical roles in the anti-osteoporosis effect of FTZ (Supplementary Figure 1). The expression of Nfatc1, one of the 77 common genes, increased after the RANKL stimulation, which activated several downstream genes related to the osteoclast lineage, including Trap and Mmp9, thus eventually leading to the formation of mature osteoclast with bone resorption function [57]. In addition, as with previous studies, our RRA analysis results confirmed the expression level of critical osteoclastogenesis genes, including Trap and Mmp9, are all significantly increased among these three GEO datasets. Therefore, we further investigated the regulatory effect of FTZ on osteoclast differentiation, bone resorption function, and the mRNA expression level of these representative osteoclast genes. We confirmed that FTZ-containing serum can downregulate these genes expression, including Nfatc1, Trap and Mmp9 and suppress osteoclast differentiation and bone resorption function (Figure 7).

GOBP and KEGG enrichment analyses were further conducted to explore and probe the functions of overlapping genes. We mainly concentrated on highly statistically significant terms related to osteoclast differentiation. And these enrichment results (Figure 4) indicated that FTZ might exert its osteoclast inhibitory effect through modulating the myeloid cell and osteoclast differentiation, inflammation, as well as TNF-α signaling pathway. Osteoclasts derive from the differentiation and fusion of mononucleated hematopoietic precursors of the myeloid lineage. Under the stimulation of RANKL, NF-κB and c-Fos activates the expression of NFATc1 that is a master transcription factor of osteoclast differentiation. Subsequently, the activated NFATc1 drives several osteoclastogenic genes, such as Trap and Mmp9 [57]. Mmp9, an extracellular matrix (ECM) consuming enzyme, expresses primarily in osteoclasts and is associated with bone resorption [58]. IL6 has been proved to accelerate osteoclastogenesis and bone resorption [59]. This might partly explain how FTZ attenuated bone loss *in vitro* and *in vivo* (Figures 7–8). Inflammation is crucial for bone remolding. As reported, inflammation in the bone microenvironment contributes to over-activated osteoclasts in osteolysis [60]. Under conditions of inflammation, TNF-α stimulates osteoclastogenesis and bone resorption in synergy with RANKL and other inflammatory cytokines such as IL6 [61]. IL6, an inflammatory cytokine, is one of the most notable increasing genes and play a critical role in the PPI network (Figure 3). The PPI (Figure 3B) and BP enrichment (Figure 4A) results showed that the regulation of the inflammatory response may contribute to the mechanism of FTZ in anti-osteoclastogenesis. Previous study has reported that FTZ can improve rabbit’s iliac artery restenosis through increasing serum adiponectin while reducing serum IL6 and TNF-α [62]. Our current ELISA result also confirmed that FTZ can reverse the increasing serum levels of TNF-α and IL6 induced by OVX (Figure 8B and 8C).

In addition to network pharmacology, molecular docking is also widely used to investigate therapeutic mechanisms of TCM [63]. Our docking results confirmed that FTZ components have good binding activities with TNF-α pathway enriched genes, such as Ptgs2, Mmp9 and Il6 (Figures 5 and 6). TNF-α is a critical cytokine to induce TNF signaling pathway via binding to two known transmembrane receptors, TNFR1 and TNFR2. TNFR1 acts as the major mediator of TNF-induced signaling pathways, which in turn stimulate two distinct pathways, NF-kB and MAPK cascades. These stimulations subsequently activate many osteoclastogenesis genes, including c-Fos, Il6 and Mmp9 [64]. The current study found that osteoclast-related genes, including Il6 and Mmp9, may be the therapeutic targets for FTZ suppressing osteoclast differentiation, and FTZ may hamper osteoclastogenesis through TNF-α signaling pathway.

Regarding the limitations of current study and the aspects that can be improved in the future, we discussed only a small proportion of potential mechanisms for FTZ to suppress osteoclast differentiation. Moreover, our validated studies only focused on limited and common osteoclast-related genes, which certainly omit other possible important mechanisms, such as its potential regulations on FoxO and PI3K-Akt signaling pathways. Therefore, further studies are required to gain deeper insight into these pathways and underlining mechanisms.

## CONCLUSIONS

This bioinformatics analysis combined with the experimental verification study illustrated the potential effects of FTZ on suppressing osteoclastogenesis and bone loss induced by OVX, through regulating the cellular response to inflammation, myeloid cell and osteoclast differentiation, and TNF-α signaling pathway. However, the current study only investigates an important portion of predicted mechanisms based on limited experiment results. More comprehensive and in-depth research would be conducted in the future.

## Supplementary Materials

Supplementary Data

Supplementary Figure 1

Supplementary Tables

Supplementary Table 3
