# Supplementary material for: The therapeutic effect of Fufang Zhenshu Tiaozhi (FTZ) on osteoclastogenesis and ovariectomized-induced bone loss: evidence from network pharmacology, molecular docking and experimental validation
Source: Aging (Albany NY). 2022 Jul 12;14(14):5727–48. doi: 10.18632/aging.204172 (PMC9365554; doi:10.18632/aging.204172)
Supplement: Supplementary Data [file aging-14-204172-s001.pdf]

SUPPLEMENTARY DATA

The raw data supporting the conclusions of present study have been uploaded to the figshare repository (https://doi.org/10.6084/m9.figshare.18666731.v1).
