# Supplementary material for: The therapeutic effect of Fufang Zhenshu Tiaozhi (FTZ) on osteoclastogenesis and ovariectomized-induced bone loss: evidence from network pharmacology, molecular docking and experimental validation
Source: Aging (Albany NY). 2022 Jul 12;14(14):5727–48. doi: 10.18632/aging.204172 (PMC9365554; doi:10.18632/aging.204172)
Supplement: Supplementary Figure 1 [file aging-14-204172-s002.pdf]

SUPPLEMENTARY FIGURE

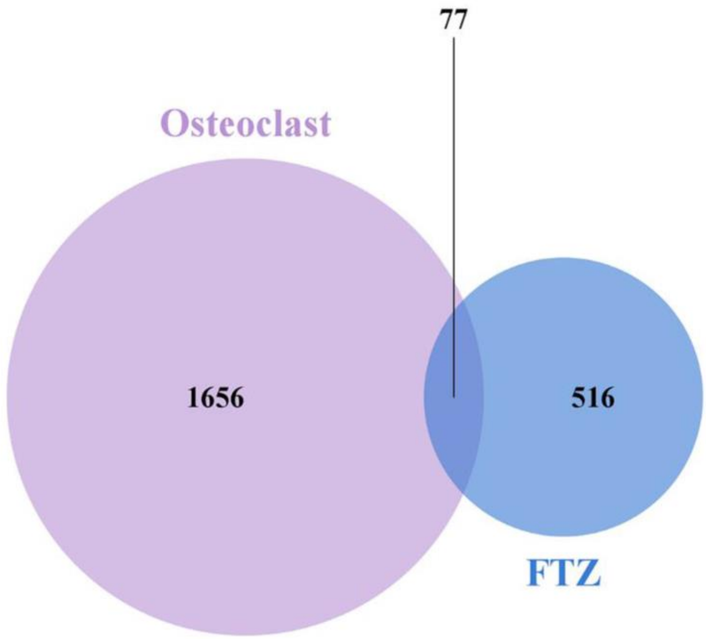

Supplementary Figure 1. The Venn diagram presented the targets of FTZ and DEGs of osteoclast differentiation. 77 overlapping targets represent the potential therapeutic genes for FTZ exerting its inhibitory effect.
