# Supplementary material for: The therapeutic effect of Fufang Zhenshu Tiaozhi (FTZ) on osteoclastogenesis and ovariectomized-induced bone loss: evidence from network pharmacology, molecular docking and experimental validation
Source: Aging (Albany NY). 2022 Jul 12;14(14):5727–48. doi: 10.18632/aging.204172 (PMC9365554; doi:10.18632/aging.204172)
Supplement: Supplementary Tables [file aging-14-204172-s003.pdf]

SUPPLEMENTARY TABLES

Supplementary Table 1. The expression profile of 52 Robust DEGs.

Gene	*P*.Value	Freq	Condition
MMP9	5.69E-06	3	Up
ACP5	0.000024	3	Up
CTSK	6.32E-05	3	Up
TRAF1	0.000779	3	Up
GSTT1	0.003896	3	Up
CCR1	0.003896	2	Up
IL15	0.007207	2	Up
CCL12	0.008563	1	Up
VEGFC	0.013889	3	Up
BPNT1	0.01711	1	Up
SAA3	0.01711	1	Up
IL6	0.018235	2	Up
SCIN	0.018342	3	Up
SLC9B2	0.01899	2	Up
FPR1	0.025641	1	Up
OSCAR	0.025641	1	Up
SLC6A8	0.02645	2	Up
CHCHD10	0.030144	2	Up
SERPINB2	0.034155	1	Up
SLC6A4	0.034155	1	Up
MYCL	0.039296	2	Up
PACRG	0.041486	2	Up
SELP	0.042653	2	Up
MARCO	0.042653	1	Up
CCL5	0.044876	2	Up
RNASE4	0.001033	2	Down
LSP1	0.005672	3	Down
DAB2	0.007035	2	Down
PROS1	0.00796	3	Down
RASGRP3	0.009164	2	Down
LY86	0.009434	3	Down
HPGD	0.009934	2	Down
MS4A6B	0.011566	2	Down
IFIT1	0.012081	1	Down
KLK1B11	0.012081	1	Down
IGHM	0.012081	1	Down
MAFB	0.015191	3	Down
MS4A6C	0.015191	2	Down
GPR65	0.022679	2	Down
CFH	0.023615	3	Down
SLC7A7	0.024129	1	Down
P2RY6	0.024129	1	Down
IRF8	0.027602	2	Down
ITGAM	0.028905	2	Down
DYNLT1B	0.036144	1	Down

FCGR1	0.036144	2	Down
APOE	0.036144	1	Down
MPEG1	0.040357	2	Down
CLEC7A	0.04676	2	Down
SQRDL	0.048127	1	Down
RNF144B	0.048127	1	Down
IGHJ1	0.048127	1	Down

Supplementary Table 2. The expression profile of 77 common targets among three datasets.

Gene	GSE21639	GSE54779	GSE74847	Mean logfc	Gene Feature
CD38	/	4.72915	/	4.72915	Up
MMP9	3.89517	3.92673	6.17478	4.66556	Up
RORC	/	/	3.88164	3.88164	Up
CCR1	2.46905	/	5.10388	3.78647	Up
MET	/	3.39635	/	3.39635	Up
SELP	1.41663	/	5.30075	3.35869	Up
SLC7A11	/	3.09128	/	3.09128	Up
IL1B	/	2.69341	/	2.69341	Up
IL6	2.07554	3.06137	/	2.56846	Up
HPGD	/	−2.22487	−2.80545	−2.51516	Down
PTGS1	/	/	−2.48925	−2.48925	Down
MST1R	1.14386	/	3.63957	2.39171	Up
ALOX5	/	/	−2.30180	−2.30180	Down
PTAFR	/	/	−2.27864	−2.27864	Down
ALOX5AP	/	/	−2.24446	−2.24446	Down
CYSLTR1	/	/	−2.19371	−2.19371	Down
PTGES	/	2.19322	/	2.19322	Up
ABCG2	2.11812	/	/	2.11812	Up
GPR55	/	/	2.04992	2.04992	Up
LGALS9	−2.20479	/	−1.81709	−2.01094	Down
PAWR	1.99516	/	/	1.99516	Up
ALB	1.98913	/	/	1.98913	Up
PLA2G2A	1.98572	/	/	1.98572	Up
RPS6KB2	−1.97837	/	/	−1.97837	Down
PPARG	/	−1.88844	/	−1.88844	Down
PFKFB3	/	1.78137	/	1.78137	Up
PTGER4	−1.75368	/	/	−1.75368	Down
OGFRL1	/	/	−1.72803	−1.72803	Down
NOS2	/	/	1.67172	1.67172	Up
RARB	/	/	1.64799	1.64799	Up
SCN9A	1.64047	/	/	1.64047	Up
NQO1	/	−1.63444	/	−1.63444	Down
F10	/	1.61736	/	1.61736	Up
KDM5B	/	/	1.61537	1.61537	Up
KCNA3	/	/	−1.59066	−1.59066	Down
NR1H3	/	1.53253	/	1.53253	Up
FABP4	/	1.51528	/	1.51528	Up
CCND1	/	1.50698	/	1.50698	Up
KDM5A	1.48772	/	/	1.48772	Up

FABP5	/	1.46628	/	1.46628	Up
IL6ST	/	−1.45754	/	−1.45754	Down
HSD11B1	/	1.45338	/	1.45338	Up
ODC1	/	1.44912	/	1.44912	Up
PPARD	1.44155	/	/	1.44155	Up
DHFR	/	1.38862	/	1.38862	Up
FABP1	−1.38055	/	/	−1.38055	Down
PLK3	/	1.35433	/	1.35433	Up
CDC25A	/	1.34483	/	1.34483	Up
SERPINE1	1.33711	/	/	1.33711	Up
PIK3CB	/	/	1.29386	1.29386	Up
SLC16A3	/	1.28471	/	1.28471	Up
IGFBP4	/	/	−1.28140	−1.28140	Down
ADORA2A	/	1.27297	/	1.27297	Up
HBEGF	/	/	1.26051	1.26051	Up
MPO	/	1.25160	/	1.25160	Up
CHEK1	/	1.24099	/	1.24099	Up
IL10	/	1.23120	/	1.23120	Up
P4HA1	/	1.22795	/	1.22795	Up
EDNRB	/	−1.20335	/	−1.20335	Down
PTGIR	/	/	−1.19760	−1.19760	Down
DHCR7	/	/	−1.14070	−1.14070	Down
NDUFC2	1.13906	/	/	1.13906	Up
HSPD1	/	1.12305	/	1.12305	Up
CXCR3	/	−1.11033	/	−1.11033	Down
PTPRC	−1.10211	/	/	−1.10211	Down
HIF1A	/	1.09087	/	1.09087	Up
PIK3CG	/	/	−1.08327	−1.08327	Down
IGFBP1	−1.08243	/	/	−1.08243	Down
CYCS	/	1.07060	/	1.07060	Up
RXRG	−1.06722	/	/	−1.06722	Down
ALDH2	/	−1.05327	/	−1.05327	Down
ERAP1	/	/	−1.02507	−1.02507	Down
FOS	/	/	1.01127	1.01127	Up
RXRA	/	/	−1.00292	−1.00292	Down
PDCD4	/	−2.02460	1.64341	−0.19060	Down
PTGS2	−1.08952	2.57901	−1.12126	0.12274	Up
BCL2L1	−1.03134	1.07730	/	0.02298	Up
