# Supplementary material for: The therapeutic effect of Fufang Zhenshu Tiaozhi (FTZ) on osteoclastogenesis and ovariectomized-induced bone loss: evidence from network pharmacology, molecular docking and experimental validation
Source: Aging (Albany NY). 2022 Jul 12;14(14):5727–48. doi: 10.18632/aging.204172 (PMC9365554; doi:10.18632/aging.204172)
Supplement: Supplementary Table 3 [file aging-14-204172-s004.docx]

**Supplementary Table 3. Summary of all docking results.**

| **Receptor** | **PDB ID** | **Ligand** | **mode** | **Affinity(kcal/mol)** |
| --- | --- | --- | --- | --- |
| PTGS2 | 1CVU | Coptisine | 1 | -10.3 |
| PIK3CB | 2Y3A | Coptisine | 1 | -9.9 |
| PTGS2 | 1CVU | Magnoflorine | 1 | -9.7 |
| MMP9 | 6ESM | Coptisine | 1 | -9.5 |
| PTGS2 | 1CVU | 13-Methylberberine | 1 | -9.5 |
| IL1B | 3LTQ | GinsenosideF1 | 1 | -9.4 |
| PTGS2 | 1CVU | Thalifendine | 1 | -9.4 |
| PTGS2 | 1CVU | Berberine | 1 | -9.3 |
| PTGS2 | 1CVU | GinsenosideRg1 | 1 | -9.3 |
| PIK3CB | 2Y3A | Thalifendine | 1 | -9.2 |
| PIK3CB | 2Y3A | GinsenosideRd | 1 | -9.1 |
| MMP9 | 6ESM | Salidroside | 1 | -9 |
| MMP9 | 6ESM | GinsenosideRd | 1 | -8.9 |
| PTGS2 | 1CVU | 10-Hydroxyoleoside | 1 | -8.9 |
| PTGS2 | 1CVU | Epiberberine | 1 | -8.9 |
| MMP9 | 6ESM | Epiberberine | 1 | -8.7 |
| MMP9 | 6ESM | GinsenosideF1 | 1 | -8.7 |
| PTGS2 | 1CVU | Columbamine | 1 | -8.7 |
| PTGS2 | 1CVU | Jatrorrhizine | 1 | -8.7 |
| IL1B | 3LTQ | GinsenosideRd | 1 | -8.6 |
| IL1B | 3LTQ | NotoginsenosideR1 | 1 | -8.6 |
| MMP9 | 6ESM | Berberine | 1 | -8.6 |
| MMP9 | 6ESM | GinsenosideRg1 | 1 | -8.6 |
| MMP9 | 6ESM | NotoginsenosideR1 | 1 | -8.6 |
| PIK3CB | 2Y3A | Epiberberine | 1 | -8.6 |
| PIK3CB | 2Y3A | GinsenosideF1 | 1 | -8.6 |
| PTGS2 | 1CVU | Palmatine | 1 | -8.6 |
| IL6 | 1ALU | GinsenosideRd | 1 | -8.5 |
| PTGS2 | 1CVU | dimethylester | 1 | -8.5 |
| PTGS2 | 1CVU | Hydroxylpalmatine | 1 | -8.5 |
| MMP9 | 6ESM | Pinoresinol | 1 | -8.4 |
| IL1B | 3LTQ | Coptisine | 1 | -8.2 |
| PIK3CB | 2Y3A | Berberine | 1 | -8.2 |
| PIK3CB | 2Y3A | Oleanolicacid | 1 | -8.2 |
| IL1B | 3LTQ | Pomolicacid | 1 | -8.1 |
| MMP9 | 6ESM | Columbamine | 1 | -8.1 |
| PIK3CB | 2Y3A | GinsenosideRg1 | 1 | -8.1 |
| IL1B | 3LTQ | Epiberberine | 1 | -8 |
| PIK3CB | 2Y3A | 13-Methylberberine | 1 | -8 |
| PIK3CB | 2Y3A | Columbamine | 1 | -8 |
| PIK3CB | 2Y3A | Hydroxylpalmatine | 1 | -8 |
| PIK3CB | 2Y3A | NotoginsenosideR1 | 1 | -8 |
| MMP9 | 6ESM | Oleanolicacid | 1 | -7.9 |
| PTGS2 | 1CVU | Salidroside | 1 | -7.9 |
| IL1B | 3LTQ | Thalifendine | 1 | -7.8 |
| MMP9 | 6ESM | 13-Methylberberine | 1 | -7.8 |
| PIK3CB | 2Y3A | Masilinicacid | 1 | -7.8 |
| PTGS2 | 1CVU | Pinoresinol | 1 | -7.8 |
| MMP9 | 6ESM | Masilinicacid | 1 | -7.7 |
| PIK3CB | 2Y3A | GinsenosideRb1 | 1 | -7.7 |
| IL6 | 1ALU | GinsenosideRb1 | 1 | -7.6 |
| MMP9 | 6ESM | Thalifendine | 1 | -7.6 |
| PIK3CB | 2Y3A | Pinoresinol | 1 | -7.6 |
| PTGS2 | 1CVU | Dehydrocorydaline | 1 | -7.6 |
| IL1B | 3LTQ | Masilinicacid | 1 | -7.5 |
| IL1B | 3LTQ | Oleanolicacid | 1 | -7.5 |
| IL6 | 1ALU | NotoginsenosideR1 | 1 | -7.5 |
| MMP9 | 6ESM | GinsenosideRb1 | 1 | -7.5 |
| MMP9 | 6ESM | Pomolicacid | 1 | -7.5 |
| PIK3CB | 2Y3A | Jatrorrhizine | 1 | -7.5 |
| PIK3CB | 2Y3A | Magnoflorine | 1 | -7.5 |
| PIK3CB | 2Y3A | Palmatine | 1 | -7.5 |
| IL1B | 3LTQ | GinsenosideRg1 | 1 | -7.4 |
| IL1B | 3LTQ | Palmatine | 1 | -7.4 |
| IL1B | 3LTQ | Berberine | 1 | -7.3 |
| IL1B | 3LTQ | Jatrorrhizine | 1 | -7.3 |
| IL6 | 1ALU | GinsenosideF1 | 1 | -7.3 |
| MMP9 | 6ESM | Hydroxylpalmatine | 1 | -7.3 |
| PIK3CB | 2Y3A | Dehydrocorydaline | 1 | -7.3 |
| PIK3CB | 2Y3A | dimethylester | 1 | -7.3 |
| PIK3CB | 2Y3A | Salidroside | 1 | -7.3 |
| IL6 | 1ALU | Masilinicacid | 1 | -7.2 |
| MMP9 | 6ESM | dimethylester | 1 | -7.2 |
| MMP9 | 6ESM | Jatrorrhizine | 1 | -7.2 |
| MMP9 | 6ESM | Magnoflorine | 1 | -7.2 |
| IL6 | 1ALU | Oleanolicacid | 1 | -7.1 |
| IL1B | 3LTQ | Columbamine | 1 | -7 |
| IL1B | 3LTQ | GinsenosideRb1 | 1 | -7 |
| IL1B | 3LTQ | Pinoresinol | 1 | -7 |
| IL6 | 1ALU | Pomolicacid | 1 | -7 |
| MMP9 | 6ESM | Palmatine | 1 | -7 |
| PIK3CB | 2Y3A | Pomolicacid | 1 | -7 |
| PTGS2 | 1CVU | 57-Dimethoxycoumarin | 1 | -7 |
| IL1B | 3LTQ | 13-Methylberberine | 1 | -6.9 |
| IL1B | 3LTQ | Salidroside | 1 | -6.9 |
| IL6 | 1ALU | Magnoflorine | 1 | -6.9 |
| MMP9 | 6ESM | Dehydrocorydaline | 1 | -6.9 |
| IL6 | 1ALU | Thalifendine | 1 | -6.8 |
| IL1B | 3LTQ | 10-Hydroxyoleoside | 1 | -6.7 |
| IL6 | 1ALU | Pinoresinol | 1 | -6.7 |
| MMP9 | 6ESM | 10-Hydroxyoleoside | 1 | -6.7 |
| IL1B | 3LTQ | Dehydrocorydaline | 1 | -6.6 |
| IL1B | 3LTQ | Magnoflorine | 1 | -6.6 |
| MMP9 | 6ESM | 57-Dimethoxycoumarin | 1 | -6.6 |
| IL1B | 3LTQ | Hydroxylpalmatine | 1 | -6.5 |
| IL6 | 1ALU | Coptisine | 1 | -6.5 |
| PIK3CB | 2Y3A | 10-Hydroxyoleoside | 1 | -6.5 |
| MMP9 | 6ESM | Protocatechuicacid | 1 | -6.4 |
| IL6 | 1ALU | Jatrorrhizine | 1 | -6.3 |
| IL6 | 1ALU | Salidroside | 1 | -6.2 |
| IL6 | 1ALU | Berberine | 1 | -6.1 |
| IL6 | 1ALU | Epiberberine | 1 | -6.1 |
| IL6 | 1ALU | GinsenosideRg1 | 1 | -6 |
| IL1B | 3LTQ | dimethylester | 1 | -5.9 |
| IL6 | 1ALU | Hydroxylpalmatine | 1 | -5.9 |
| MMP9 | 6ESM | Eucommiol | 1 | -5.9 |
| PTGS2 | 1CVU | Protocatechuicacid | 1 | -5.9 |
| IL1B | 3LTQ | Protocatechuicacid | 1 | -5.8 |
| IL6 | 1ALU | 13-Methylberberine | 1 | -5.8 |
| PTGS2 | 1CVU | Eucommiol | 1 | -5.8 |
| IL6 | 1ALU | Columbamine | 1 | -5.7 |
| IL6 | 1ALU | Palmatine | 1 | -5.7 |
| IL6 | 1ALU | Protocatechuicacid | 1 | -5.7 |
| IL1B | 3LTQ | 57-Dimethoxycoumarin | 1 | -5.6 |
| IL6 | 1ALU | 10-Hydroxyoleoside | 1 | -5.6 |
| IL6 | 1ALU | 57-Dimethoxycoumarin | 1 | -5.6 |
| IL6 | 1ALU | Dehydrocorydaline | 1 | -5.6 |
| IL6 | 1ALU | dimethylester | 1 | -5.6 |
| PIK3CB | 2Y3A | 57-Dimethoxycoumarin | 1 | -5.5 |
| IL1B | 3LTQ | Eucommiol | 1 | -5.2 |
| IL6 | 1ALU | Eucommiol | 1 | -5 |
| PIK3CB | 2Y3A | Protocatechuicacid | 1 | -5 |
| PIK3CB | 2Y3A | Eucommiol | 1 | -4.8 |
| PTGS2 | 1CVU | Oleanolicacid | 1 | -1.7 |
| PTGS2 | 1CVU | Masilinicacid | 1 | -0.2 |
| PTGS2 | 1CVU | GinsenosideF1 | 1 | 1.5 |
| PTGS2 | 1CVU | Pomolicacid | 1 | 2.4 |
| PTGS2 | 1CVU | NotoginsenosideR1 | 1 | 12.9 |
| PTGS2 | 1CVU | GinsenosideRd | 1 | 14.3 |
| PTGS2 | 1CVU | GinsenosideRb1 | 1 | 18.9 |
